# 3D Finite Element Analysis of a Concrete Dam Behavior under Changing Hydrostatic Load: A Case Study

**DOI:** 10.3390/ma15030921

**Published:** 2022-01-25

**Authors:** Pavel Žvanut

**Affiliations:** Slovenian National Building and Civil Engineering Institute, Dimičeva 12, 1000 Ljubljana, Slovenia; pavel.zvanut@zag.si; Tel.: +386-1-2804-491

**Keywords:** concrete dam, finite element method, material properties, structure behavior, measuring instruments, monitoring, time series analysis, horizontal displacements, hydrostatic pressure

## Abstract

In this study, a large arch-gravity Moste Dam was analyzed, where an automated system for the measurements of horizontal displacements of the upper part of the dam was established. Two-dimensional (2D) and three-dimensional (3D) analyses of dam behavior, taking into account the earth pressures and the hydrostatic load, using the finite element method (FEM)-based computer program DIANA, were performed. The influence of lowering the water level of the reservoir by 6.2 m, on the horizontal displacements of the upper part of the dam, at stationary temperature conditions, was investigated. It was found that the results of the performed 2D and 3D FEM analyses fitted in very well with the result of experimentally determined measurement of horizontal displacements (which was 0.48 mm in the upstream direction) that was obtained using a hanging pendulum. An additional comparison of the results of 3D calculations showed that the finite element mesh density had a small effect on the calculated horizontal displacements.

## 1. Introduction

Recently, the monitoring of static and dynamic behavior of complex building structures, which also include large dams, has become very important, as by closely monitoring of their behavior, we can also determine their safety. In addition to the temperature load, the hydrostatic pressure causes high stresses in large arch-gravity concrete dam structures and has consequently great influence on cracks and dam deformation [1]. Accurate and reliable prediction of dam deformation is of great importance to ensure the safe and stable operation of dams [2]. Due to climate change, the frequent extreme rainfall can cause a sudden rise of the water level of the reservoir, which can endanger the dam safety [3].

In the 1990s, Leger et al. [4,5] presented a methodology, which was based on the FEM and which could be used to determine the deformation behavior of concrete gravity dams. Using this methodology, the stress-strain state of concrete dams was much better analyzed, both in the initial design phase [6,7], as well as in the later phase of monitoring the behavior of dams [8,9]. A large amount of research about this topic, where the effects of changing hydrostatic pressure on horizontal displacements of concrete dams were taken into account, has been carried out recently. Some of researchers focusing on the detailed dealing with the displacement response of a concrete arch dam to reservoir level rise based on conventional geodetic measurements and FEM analyses during the first filling period [10], some others discussing with displacement study based on precision geodetic monitoring and numerical modeling [11,12], whereas other researchers analyzed displacement responses of the dam based on both geodetic and pendulum monitoring records [13]. Some researchers used the results of measurements with pendulums for prediction the deformation of super-high arch dam by deformation model [14], whereas the others for interpretation of concrete dam behavior with artificial neural network [15]. Some other authors have discussed the development of new numerical models, such as hydrostatic, temperature, time-displacement model for concrete dams [16]. Some researchers have focused on detailed studies of displacements dealing with diagnostic analysis of concrete dams based on seasonal hydrostatic loading [17]. Some reports have discussed actual working performance in the case of super-high arch dams [18]. Colombo et al. [19] presented a methodology to validate the finite element models of existing concrete dams with the monitoring data, whereas Li et al. [20] reviewed a literature about dam monitoring data analysis methods, which is very important for monitoring dam safety. Konakoglu et al. [21] investigated the deformation of a concrete dam during the change of the water level of the reservoir, by means of the Global Navigation Satellite Systems data. Qin et al. [22] and Yang et al. [23] separated the hydrostatic pressure component of displacements from the measured data of high concrete dams in operating conditions and calculated the deformation using the FEM analyses. Considering the generally recognized three components of dam displacement (hydraulic, seasonal and time) Wang et al. [24] proposed state space model, which is useful to improve ability of the separated displacement components of concrete dams and which was verified by an engineering example.

In the last decade there were attempts to obtain high-quality 3D models of surface geometry, based on terrestrial laser scanner (TLS) measurements, which were applied to different complex and large structures [25,26]. There were also some studies on the use of TLS measurements to monitor the deformations of hydraulic structures [27], but in this case very accurate instruments and very reliable measurements are needed.

However, in the above-mentioned articles there seems to be no exhaustive data on accurate measurements and 3D calculations of horizontal displacements of the upper part of the dam solely because of changes in hydrostatic pressure (i.e., at stationary temperature conditions), and at the same time there is no comprehensive data where an accurate 3D model of the dam would be made using contemporary measurements (e.g., TLS measurements). Consideration of changes in hydrostatic pressure is important because it represents one of the three parameters (in addition to seasonal temperature changes and time effects) that affect the horizontal displacements of a concrete dam over a long period of time. In the case of a rapid decrease of the water level of the reservoir (when the influence of the other two factors on the displacements is negligible), the horizontal displacements due to the reduction of hydrostatic pressure, which occur in a very short period of time, can be accurately evaluated.

In this article, the author presents a case study of the Moste Dam, where the reduction of the hydrostatic pressure on the arch-gravity concrete dam, on horizontal displacements of the upper part of the dam, in a very short period of time, i.e., at stationary temperature conditions, was investigated. An automated system for measurements of horizontal displacements by hanging pendulum was established in a central cross-section of the dam, and FEM analyses of a dam deformation behavior were performed. In addition to the 2D analyses, the 3D analyses were also carried out, where a very accurate geometric model of the dam was made using precise TLS measurements at reduced water level of the reservoir.

## 2. Materials and Methods

### 2.1. Stress-Strain Analysis of a Solid Body

#### 2.1.1. Basic Equations

The stress-strain state of a solid body is determined by the three groups of basic equations (equilibrium, kinematic and constitutive), which are described and explained in detail also in Žvanut [28].

The first group of basic equations represents the equilibrium equations of a rigid body, which connect the specific surface load (*p_n_*) and the specific volume load (*ν*) of the discussed body with stresses (*σ_ij_*). There are three partial differential equations for six independent scalar functions of stresses. A special solution of these equations is obtained by taking into account the boundary conditions, which require that every particle at the boundary surface of the body, on which the external load is prescribed (*S_p_*), is also in equilibrium. The equilibrium of moments is satisfied by considering the symmetry of the shear stresses. The equilibrium equations and the associated boundary conditions can be briefly written with the following equations

(1) $$\underset{i}{\sum }\frac{\partial \sigma_{ij}}{\partial x_{i}}+v_{j}=0\;\mathrm{and}\;p_{nj}=\underset{i}{\sum }\sigma_{ij}e_{ni}\;\left(i,\;j\;=x,\;y,\;z\right).$$

The second group of basic equations consists of the kinematic equations of a deformable body, which connect the components of a symmetric tensor of small strains (*ε*) with the displacements (*u*). There are nine unknown scalar functions (i.e., six strains and three displacements) in the six partial differential equations, which are briefly written with the following equation

(2) $$\varepsilon_{ij}=\frac{1}{2}\left(\frac{\partial u_{j}}{\partial x_{i}}+\frac{\partial u_{i}}{\partial x_{j}}\right)\;\left(i,\;j\;=x,\;y,\;z\right).$$

The equations are solved at the prescribed kinematic boundary conditions, which show how the analyzed body is supported (i.e., what are the displacements and rotations on the part of the boundary surface on which the displacements and rotations are prescribed (*S_u_*)), wherein the rotations (*ω*) can be expressed by displacements (*u*)

(3) $$u_{i}=u_{i}^{r}\;and\;\omega_{i}=\omega_{i}^{r}\;\left(i\;=x,\;y,\;z\right).$$

If the displacements are known as coordinate functions, the strains can be easily calculated from Equation (2). Otherwise, if the strains are known and it is desired to calculate the displacements from Equation (2), the strains must satisfy an additional six compatibility conditions, which ensure a uniform determination of the three displacements from the six kinematic equations. The necessary and sufficient conditions for the uniformity of displacements are expressed by means of compatibility equations, which can also be written with the components of the so-called Riemann tensor of the fourth order *R*(4)

(4) $$R_{ijkl}=\frac{\partial^{2}\varepsilon_{jk}}{\partial x_{i}\partial x_{l}}+\frac{\partial^{2}\varepsilon_{il}}{\partial x_{j}\partial x_{k}}-\frac{\partial^{2}\varepsilon_{jl}}{\partial x_{i}\partial x_{k}}-\frac{\partial^{2}\varepsilon_{ik}}{\partial x_{j}\partial x_{l}}=0\;\left(i,\;j,\;k,\;l\;=x,\;y,\;z\right).$$

The dependences between stresses and strains are described by a third group of basic equations, which are called constitutive or material equations. When dealing with linearly elastic solid bodies, the relations between stresses and strains are determined by Duhamel-Neumann equations. The six linear algebraic equations are briefly written with the equation

(5) $$\sigma_{ij}=2\mu \varepsilon_{ij}+\lambda \delta_{ij}I_{1}^{\varepsilon }-\beta_{T}\delta_{ij}\Delta T\;\left(i,\;j\;=x,\;y,\;z\right).$$

The inverse form is written with the following equation

(6) $$\varepsilon_{ij}=\frac{1+v}{E}\sigma_{ij}-\frac{v}{E}\delta_{ij}I_{1}^{\sigma }+\delta_{ij}\alpha_{T}\Delta T\;(i,\;j\;=\;x,\;y,\;z),$$

where *μ* (shear modulus *G*) and *λ* are Lamé’s constants of linearly elastic isotropic material, expressed by the elastic modulus *E* and the Poisson’s ratio *ν*, *δ_ij_* the Kronecker delta, *I^ε^* the first strain invariant, *I^σ^* the first stress invariant, *β_T_* the temperature parameter, *α_T_* the thermal expansion coefficient and Δ*T* the temperature change.

The following links exist between these parameters:

(7) $$\mu =\frac{E}{2\left(1+v\right)}=G\;\mathrm{and}\;\lambda =\frac{Ev}{\left(1+v\right)\left(1-2v\right)},\;0\leq v\leq \frac{1}{2}\;,$$

(8) $$I_{1}^{\varepsilon }=\varepsilon_{xx}+\varepsilon_{yy}+\varepsilon_{zz}\;\mathrm{and}\;I_{1}^{\sigma }=\sigma_{xx}+\sigma_{yy}+\sigma_{zz},$$

(9) $$I_{1}^{\varepsilon }=\frac{1-2v}{E}I_{1}^{\sigma }+\frac{3\left(1-2v\right)}{E}\beta_{T}\Delta T\;\mathrm{and}\;\beta_{T}=\frac{E}{1-2v}\alpha_{T}.$$

#### 2.1.2. Numerical Solution of Equations

The previously written equilibrium, kinematic and constitutive equations represent the system of 15 equations for 15 unknown functions (six stresses, six strains, and three displacements) describing the stress-strain state of an isotropic, linearly elastic solid body in the case of static loading, so they are also called the fundamental equations of the theory of elasticity. In addition to the six linear algebraic constitutive equations, there are nine linear partial differential equations of the first order that need to be solved under the prescribed boundary conditions. In most cases, the boundary value problem is solved by numerical procedures in which the basic equations are satisfied only in a final set of selected discrete points of the analyzed body.

For numerical solving, FEM [29] is very commonly used, in which the relationship between nodal forces and nodal displacements is defined by the following equation

**F = KU**, (10)

where **F** is a vector of nodal forces, **K** is a stiffness matrix of the system of finite elements, and **U** is a vector of nodal displacements.

**Figure 1:**
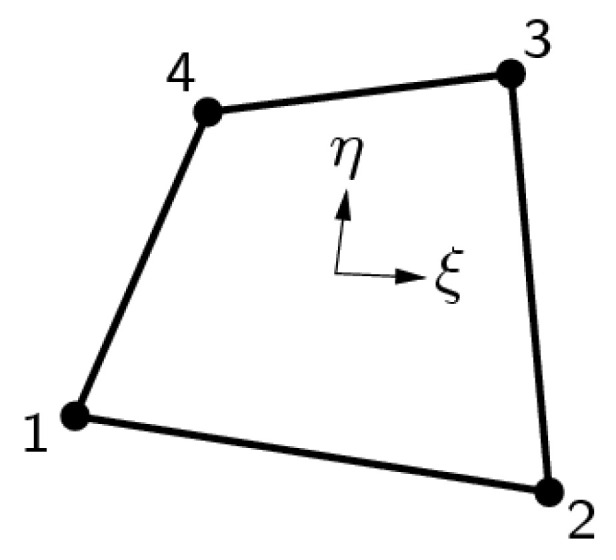
A 4-node quadrilateral isoparametric plane-strain finite element (code Q8EPS).

**Figure 2:**
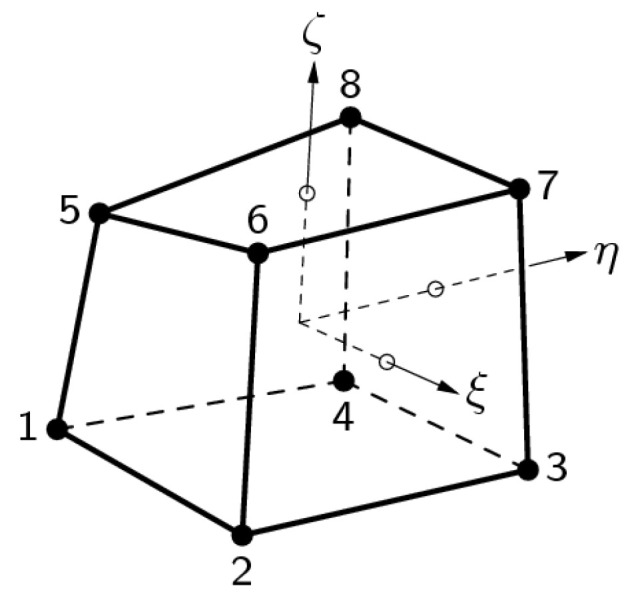
An 8-node isoparametric solid brick finite element (code HX24L).

In 2D analyses, a 4-node quadrilateral isoparametric plane-strain finite element was used (Figure 1), and in 3D analyses, an 8-node isoparametric solid brick finite element was applied (Figure 2). Both elements are based on linear interpolation according to Equations (11) and (12), and Gaussian integration [30].

The polynomial for the displacements u_x_ and u_y_ can be expressed as

(11) $$u_{i}\left(\xi ,\eta \right)=a_{0}+a_{1}\xi +a_{2}\eta +a_{3}\xi \eta \;(i=x,\;y),$$

where *a*_0_, *a*_1_, *a*_2_, *a*_3_ are the coefficients of the polynomial.

For constant shear, this polynomial yields a strain *ε*_xx_ which is constant in *x* direction and varies linearly in *y* direction and a strain *ε*_yy_ which is constant in *y* direction and varies linearly in *x* direction. The shear strain *γ*_xy_ is constant over the element area.

The polynomial for the displacements u_x_, u_y_ and u_z_ can be expressed as

(12) $$u_{i}\left(\xi ,\eta ,\zeta \right)=a_{0}+a_{1}\xi +a_{2}\eta +a_{3}\zeta +a_{4}\xi \eta +a_{5}\eta \zeta +a_{6}\xi \zeta +a_{7}\xi \eta \zeta \;(i=x,\;y,\;z),$$

where *a*_0_, *a*_1_, *a*_2_, *a*_3_, *a*_4_, *a*_5_, *a*_6_, *a*_7_ are the coefficients of the polynomial.

Typically, a rectangular brick element approximates the following strain and stress distribution over the element volume. The strain *ε_xx_* and stress *σ_xx_* are constant in *x* direction and vary linearly in *y* and *z* direction. The strain *ε_yy_* and stress *σ_yy_* are constant in *y* direction and vary linearly in *x* and *z* direction. The strain *ε*_zz_ and stress *σ_zz_* are constant in *z* direction and vary linearly in *x* and *y* direction.

#### 2.1.3. Earth Pressures and Hydrostatic Load

The determination of earth pressures and hydrostatic load on a rigid vertical retaining structure with the horizontal hinterland and the presence of water in the hinterland is described and explained in detail also in Žvanut [28].

The total active earth pressure *p_a_* (Pa) is calculated by the following equation

(13) $$p_{a}=p_{a}^{’}+p_{w},$$

where *p’_a_* the effective active earth pressure (Pa) and *p_w_* the hydrostatic pressure (Pa), which is defined by the equation

(14) $$p_{w}=\gamma_{w}h_{w}=\rho_{w}gh_{w},$$

where *γ_w_* the unit weight of water (N/m^3^), *ρ_w_* the water density (kg/m^3^), *g* the gravitational acceleration (m/s^2^) and *h_w_* the water depth (m).

The effective active earth pressure is calculated by the equation

(15) $$p_{a}^{’}=\sigma_{v}^{’}k_{a}-2c^{’}\sqrt{k_{a}}=\gamma^{\prime }hk_{a}-2c^{’}\sqrt{k_{a}},$$

where *σ’_ν_* is the effective vertical pressure (Pa), *h* the depth (m), *γ*’ the effective unit weight of soil (N/m^3^), *c’* the effective cohesion (Pa) and *k_a_* the coefficient of active earth pressure defined by the equation

(16) $$k_{a}=\frac{1-\sin\varphi^{\prime }}{1+\sin\varphi^{\prime }}=tg^{2}\left(45{}^{\circ}-\frac{\varphi^{\prime }}{2}\right)\;\mathrm{and}\;\sqrt{k_{a}}=\frac{\cos\varphi^{\prime }}{1+\sin\varphi^{\prime }},$$

where φ’ is the effective friction angle (°).

An important property of the active earth pressure is that it is smaller than the hydrostatic pressure and smaller than the earth pressure at rest, so

(17) $$k_{a}<k_{0}<1,$$

where *k_0_* is the coefficient of earth pressure at rest, which is determined for horizontal and normally consolidated soils by the equation [31]

(18) $$k_{0}=1-\sin\varphi^{\prime }\;$$

If the hinterland of the retaining structure consists of several differently solid and deformable horizontal soil layers, the active earth pressures at the boundaries of individual layers are calculated. First, the effective vertical pressures are calculated, which are then multiplied by the corresponding coefficient of active earth pressure, after that the appropriate portion of cohesion is subtracted and finally the corresponding hydrostatic pressure value is added.

The total passive earth pressure *p_p_* (Pa) is calculated by the following equation

(19) $$p_{p}=p_{p}^{’}+p_{w},$$

where *p’_p_* the effective passive earth pressure (Pa) and *p_w_* the hydrostatic pressure (Pa), which is defined by Equation (14).

The effective passive earth pressure is calculated by the equation

(20) $$p_{p}^{’}=\sigma_{v}^{’}k_{p}+2c^{’}\sqrt{k_{p}}=\gamma^{\prime }hk_{p}+2c^{’}\sqrt{k_{p}},$$

where *k_p_* is the coefficient of passive earth pressure defined by the equation

(21) $$k_{p}=\frac{1+\sin\varphi^{\prime }}{1-\sin\varphi^{\prime }}=tg^{2}\left(45{}^{\circ}+\frac{\varphi^{\prime }}{2}\right)\;\mathrm{and}\;\sqrt{k_{p}}=\frac{\cos\varphi^{\prime }}{1-\sin\varphi^{\prime }},$$

while the other parameters are explained under Equation (15).

An important property of the passive earth pressure is that it is greater than the hydrostatic pressure and greater than the earth pressure at rest, so

(22) $$k_{0}<1<k_{p}$$

If the hinterland of the retaining structure consists of several differently solid and deformable horizontal soil layers, the passive earth pressures at the boundaries of individual layers are calculated similarly to the active earth pressures. First, the effective vertical pressures are calculated, which are then multiplied by the corresponding coefficient of passive earth pressure, and finally the appropriate portion of cohesion and the corresponding hydrostatic pressure value are added.

### 2.2. Fieldwork

#### 2.2.1. Description of the Dam

**Figure 3:**
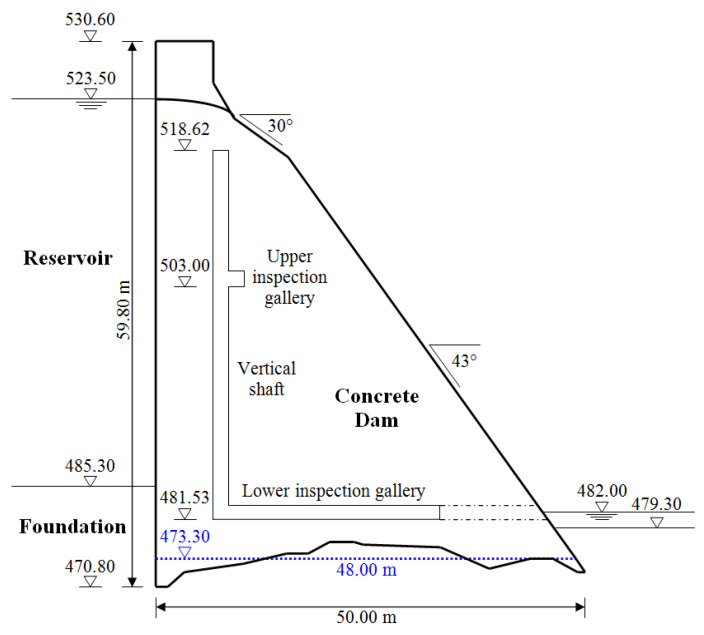
A central cross-section of the Moste Dam.

**Figure 4:**
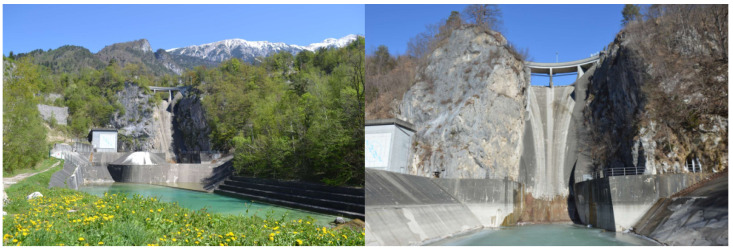
Distant and closer view of the Moste Dam from the downstream side (Photos: P. Žvanut).

A large arch-gravity concrete Moste Dam, which lies on the Sava Dolinka River in the north-western part of Slovenia, was completed in 1952. It is almost 60 m high and is the highest dam in Slovenia. It was built during the construction of the Moste hydroelectric power plant. The main characteristics of the dam are presented in Table 1 [32]. The dimensions of the central cross-section of the dam are shown in Figure 3, while the view of the Moste Dam from the downstream side, where its position in the narrow canyon is visible, is presented in Figure 4.

#### 2.2.2. Inclination Measurements

In the case of complex building structures it is very often difficult to determine the appropriate numerical values for the properties of materials (especially of the foundation soil, other soils and rocks), which means that the calculated values of individual parameters are unreliable. To determine the accordance between the calculated and actual values of individual parameters, it is necessary to monitor complex building structures [33,34], which also include large concrete dams [35,36,37]. One of the most important parameters which should be monitored at large concrete dams is the inclination of the dam structure, which is often measured by hanging pendulum. In this method, the hanging wire, which is loaded at the lowest part, serves as a basis for measuring the deviation from the vertical. If we consider the pivot point as fixed, then in the case of inclination of the dam, deviations between the wire and the surrounding points on the dam occur at displacement gauge, which are measured as a relative horizontal displacement of the pivot point according to the displacement gauge.

**Figure 5:**
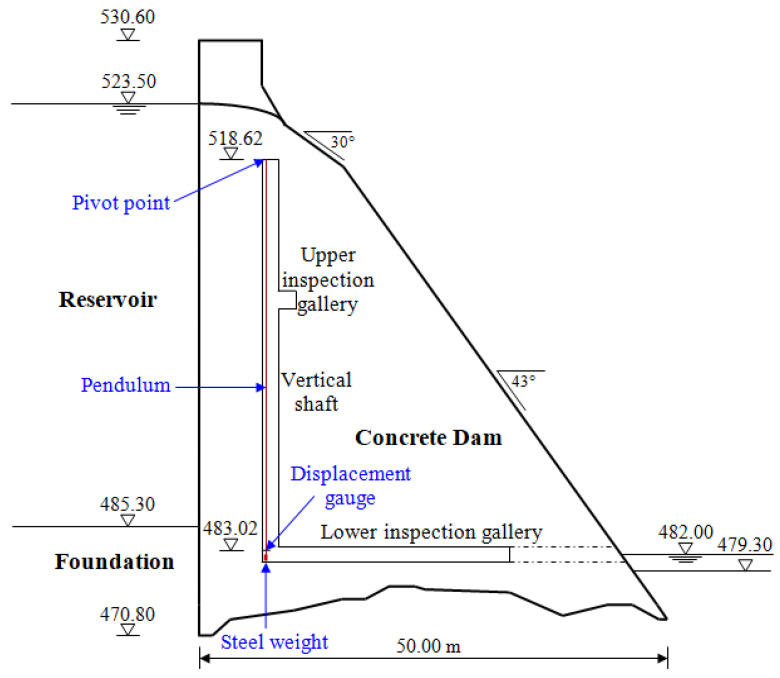
The location of a hanging pendulum in the vertical shaft.

**Figure 6:**
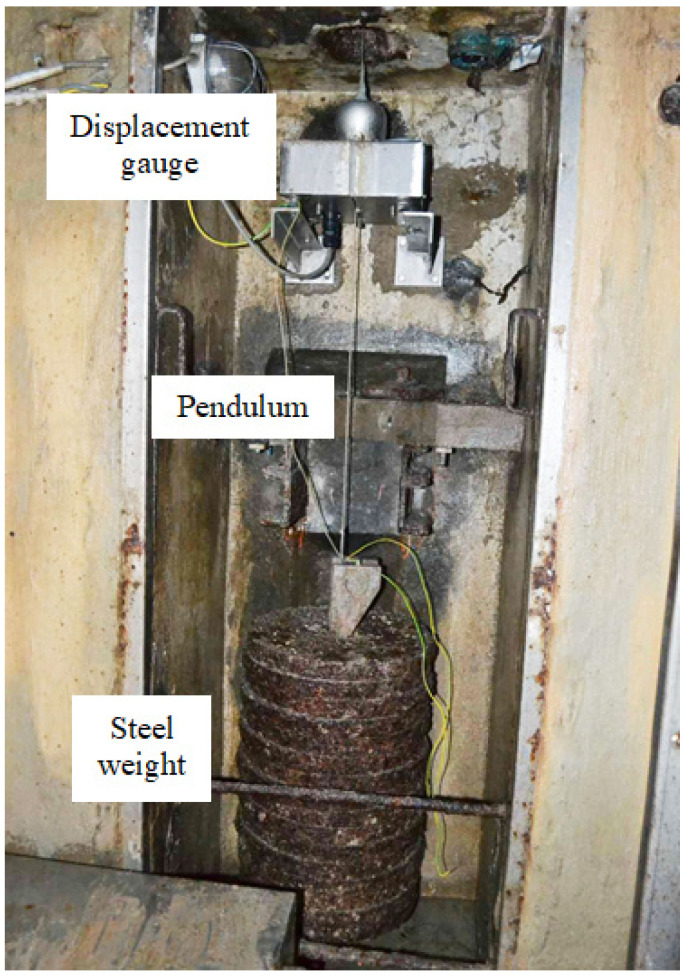
The location of a displacement gauge at the bottom of the shaft (Photo: P. Žvanut).

In the massive part of the Moste Dam, there is a one hanging pendulum, located in the vertical shaft along the upstream side of the central cross-section of the dam (Figure 5), where the typical inclination of the dam can be monitored. The top of the shaft is at altitude 518.62 m, and its bottom at altitude 481.53 m; the height of the shaft is therefore 37.09 m [38]. The pivot point of the pendulum is at the top of the shaft, while the horizontal displacement gauge is 149 cm above the bottom of the shaft (at altitude 483.02 m), above a steel weight with a mass of about 400 kg, which is hanging on a stainless steel wire with a diameter of 3 mm (Figure 6). The distance from the pivot point to the gauge is thus 35.6 m. An automatic system for measuring the horizontal displacements with a pendulum was established in 1998 [39] and upgraded in 2003 [40]. In March 2013, the three faulty inductive displacement sensors were replaced with the new ones. The results of the measurements are analyzed as part of the technical monitoring of the Moste Dam [41].

### 2.3. Procedure for the FEM Analysis

2D FEM analyses were carried out using the DIANA computer program [30], where 4-node quadrilateral isoparametric plane-strain finite elements were used in analyses. Three-dimensional (3D) FEM analyses, using 8-node isoparametric solid brick finite elements, were also performed with the same computer program, in which a 3D model of the Moste Dam was first time developed.

The mechanical properties of the mass concrete that were used to determine the stress-strain state of the dam are given in Table 2. They were determined based on data from the Report on the establishment of the technical monitoring system at the Moste Dam [42], the results of non-destructive investigations of concrete of the Moste Dam, i.e., seismic tomography and georadar [43] or obtained from other literature [4,44].

The input data for the calculation of the active earth pressures and the passive earth pressures are given in Table 3. In the computational model, the dam extends 12 m into the bedrock on the upstream side, whereas on the downstream side stretches 6 m to it. The height of sediments in the reservoir is 10 m.

## 3. Results and Discussion

### 3.1. Two-Dimensional (2D) Analyses

#### 3.1.1. Model of the Dam

**Figure 7:**
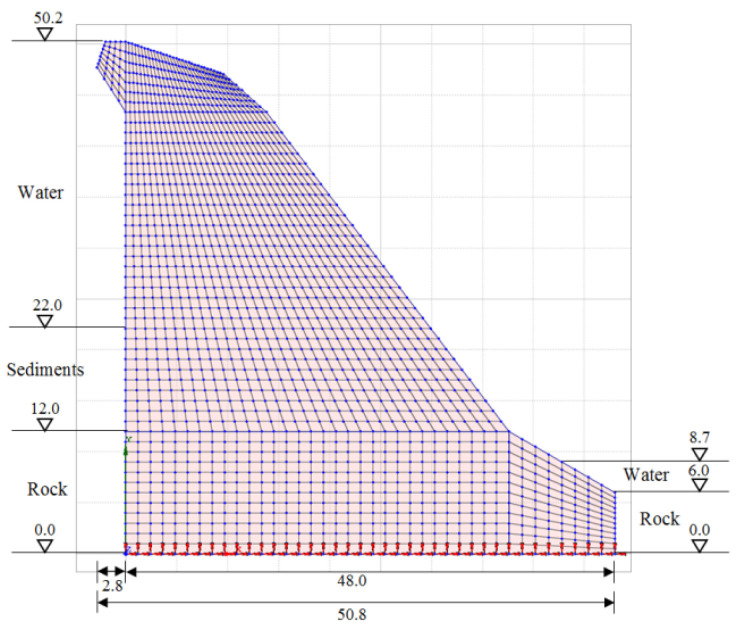
Two-dimensional (2D) model of the Moste Dam.

The geometry of the 2D model of the dam (i.e., the cross-section in the axis of the dam) was determined from the data of the Project of technical monitoring of the dam [38] and mainly from the data of TLS measurements of the Moste Dam [48,49]. The height of the discussed cross-section was 50.2 m, while the width of the cross-section was 48.0 m at the bottom of the dam (taking into account the above extension, the width was 50.8 m). The unreinforced concrete of the dam structure was modeled as an isotropic material. A linearly elastic constitutive model of the concrete behavior was used, as deformations in the range of linear elasticity were expected; the mechanical properties of the mass concrete are given in Table 2. The computational model of the plane-strain state was taken into account, so a 4-node plane-strain finite element was used in the analysis (Figure 1). The finite element mesh of the 2D model of the dam included 1674 finite elements and 1768 nodes. For boundary conditions, it was considered that the model of the dam was supported at the lower edge, which included the edges of 39 finite elements and 40 nodes; fixed nodes prevented displacements in two perpendicular directions. The flexibility of the foundation soils and the impact due to uplift pressure were assumed to be negligible. The 2D model of the dam (showing its geometry, the selected finite element mesh, and the boundary conditions; grid raster: 5 m × 5 m) is shown in Figure 7.

#### 3.1.2. Measured Horizontal Displacements

**Figure 8:**
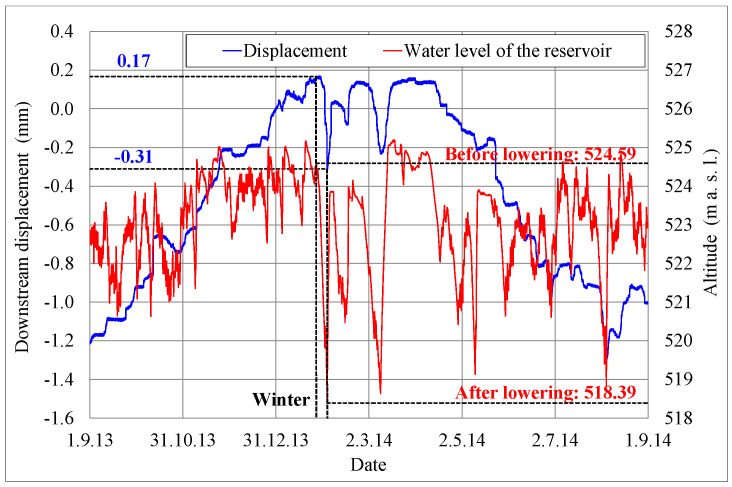
Measured displacements and the water level of the reservoir, during the analyzed year.

It is evident from Figure 8 that before lowering the water level of the reservoir (altitude 524.59 m, obtained on 27 January 2014) the measured relative displacement was 0.17 mm in the downstream direction, while after lowering the water level to an altitude of 518.39 m (obtained on 3 February 2014) the measured relative displacement was 0.31 mm in the upstream direction. The lowering of the water level by 6.2 m (at stationary temperature conditions), therefore, at the top of the vertical shaft, where the measurements were carried out, caused a relative displacement of 0.48 mm in the upstream direction.

#### 3.1.3. Calculated Horizontal Displacements

When calculating the displacements of the dam in the stream direction due to changes of the water level of the reservoir (at stationary temperature conditions), the total active earth pressures on the upstream side of the dam (due to water in the reservoir, sediments and rock; Table 4) and the total passive earth pressures on the downstream side of the dam (due to water in the stilling basin and rock; Table 5) were considered to be a load of the dam. The lowering of the water level of the reservoir from an altitude of 524.59 m to an altitude of 518.39 m resulted in a reduction of the total active earth pressures on the dam (Table 4).

**Figure 9:**
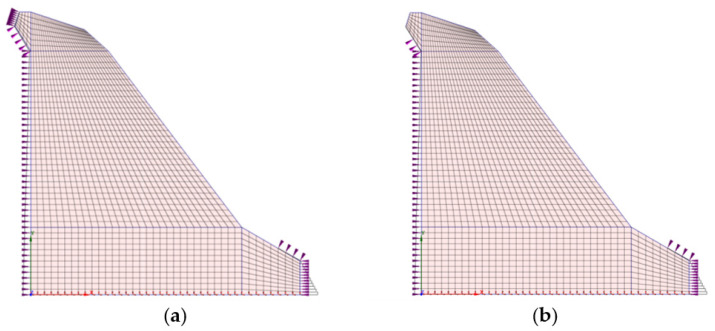
Loading of the dam according to the water level of the reservoir: (**a**) before lowering and (**b**) after lowering.

**Figure 10:**
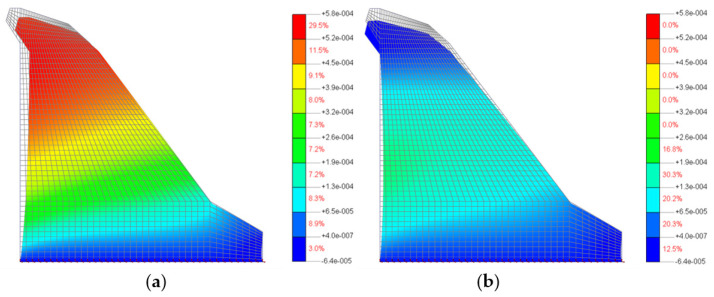
Horizontal displacements of the dam according to the water level of the reservoir: (**a**) before lowering and (**b**) after lowering.

Figure 9 shows the loading of the 2D model of the Moste Dam before lowering the water level of the reservoir, which was 524.59 m a.s.l., and after lowering the water level of the reservoir, which was 518.39 m a.s.l., while Figure 10 shows the calculated horizontal displacements of the 2D model of the Moste Dam in both mentioned cases (the displacement scale is significantly larger than the scale in which the dam is shown, because otherwise the actual displacements of up to a few mm would not be observed at all with such a large dam; the magnification factor of 5000 was considered for displacements). The results of calculations of horizontal displacements on the 2D model show that in the case of full water reservoir, the upper part of the dam moves in the downstream direction, while after lowering the water level the upper part of the dam moves upstream, i.e., in the opposite direction (the calculated upstream horizontal displacement, due to the lowering of the water level, was 0.49 mm at the top of the vertical shaft).

Comparison with the measured horizontal displacement, which was 0.48 mm (see Section 3.1.2), shows an excellent match between the measured and calculated value of the upstream horizontal displacement of the dam, at the top of the vertical shaft, due to lowering the water level of the reservoir (at stationary temperature conditions).

### 3.2. Three-Dimensional (3D) Analyses

#### 3.2.1. Model of the Dam

**Figure 11:**
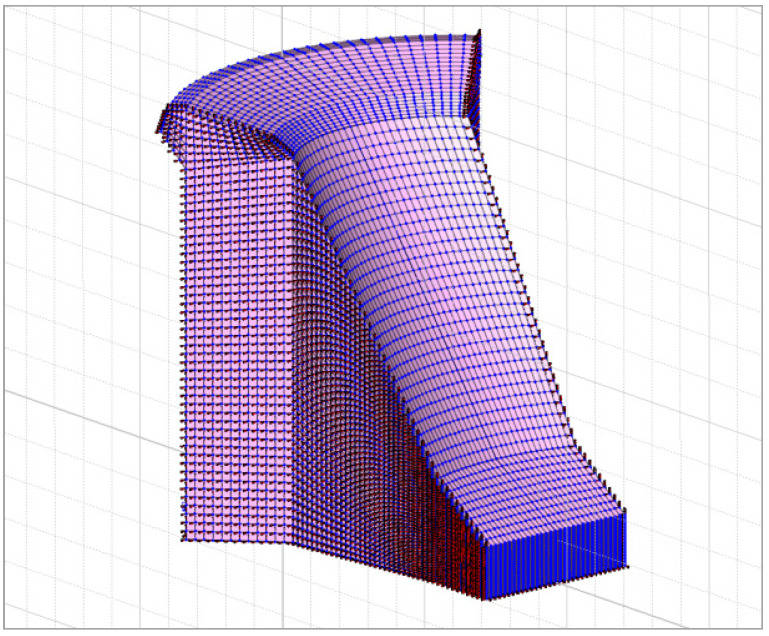
Three-dimensional (3D) model of the Moste Dam.

The geometry of the 3D model of the dam (i.e., the entire dam) was determined from the data of the Project of technical monitoring of the dam [38], from the geometry of the 2D model of the dam (Section 3.1.1) and mainly from the data of TLS measurements of the Moste Dam [48]. The height and width of the discussed model were the same as in the 2D model, while the depth was 42.4 m. As with the 2D model, the isotropic properties of the concrete, the same mechanical properties and the linearly elastic material model were taken into account. The computational model of the solid was considered, so an 8-node isoparametric solid brick finite element was used in the analysis (Figure 2). In the process of making the 3D model, the basic 2D geometry was first divided into quadrilaterals, which were later upgraded to octagons. The finite element mesh of the 3D model of the dam included 72,000 finite elements and 77,748 nodes. For boundary conditions, it was considered that the model of the dam was supported at the bottom, which included 1560 finite elements and 1643 nodes (in the nodes, displacements in three perpendicular directions are disabled) and on the sides of the dam, which included the surfaces of 4800 finite elements and 5016 nodes (in the nodes, displacements in two perpendicular directions are disabled; the exception is the stream direction). It was assumed that the settlements of the foundation soils and the impact due to uplift pressure were negligible. In the calculations, it was found that increasing the density of the finite element mesh does not affect the results. The 3D model of the dam (showing its geometry, the selected finite element mesh, and the boundary conditions; grid raster: 5 m × 5 m) is shown in Figure 11.

#### 3.2.2. Calculated Horizontal Displacements

The magnitude of the measured displacement was determined in Section 3.1.2, where it was found that when lowering the water level of the reservoir from an altitude of 524.59 m a.s.l. to an altitude of 518.39 m a.s.l. (i.e., by 6.2 m), at stationary temperature conditions, the measured horizontal displacement at the top of the vertical shaft was 0.48 mm in the upstream direction.

The load of the dam, which was taken into account when calculating the displacements of the dam in the stream direction due to changes in hydrostatic pressure, was determined in Section 3.1.3.

**Figure 12:**
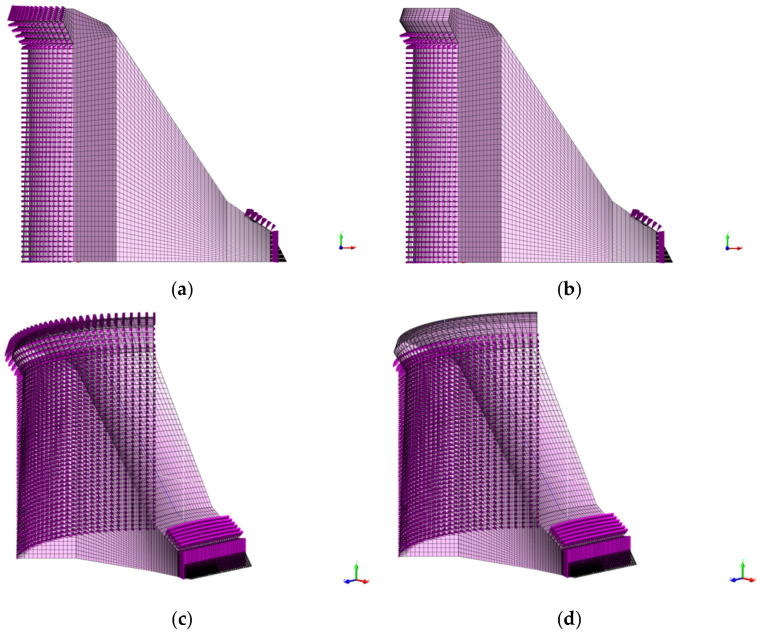
Loading of the dam according to the water level of the reservoir: (**a**) before lowering—view in the z-axis direction, (**b**) after lowering—view in the z-axis direction, (**c**) before lowering—isometric view and (**d**) after lowering—isometric view.

**Figure 13:**
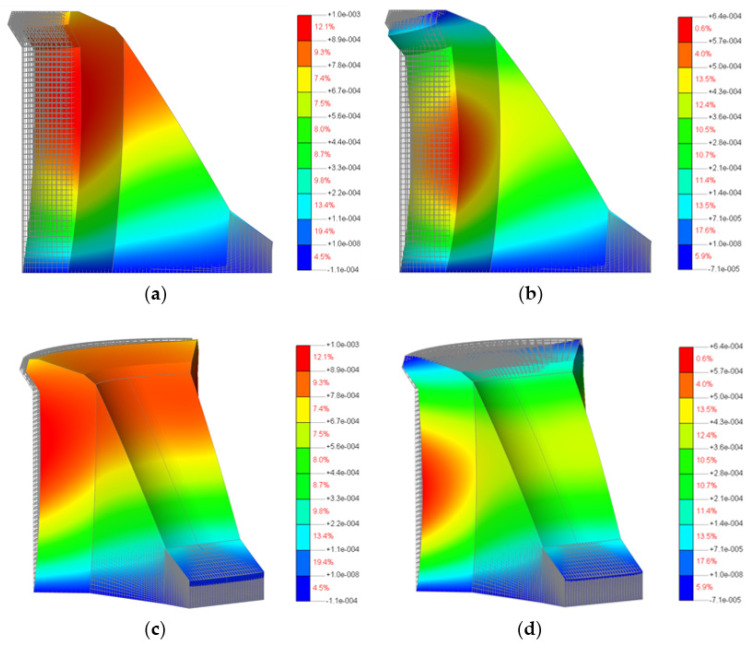
Horizontal displacements of the dam according to the water level of the reservoir: (**a**) before lowering—view in the z-axis direction, (**b**) after lowering—view in the z-axis direction, (**c**) before lowering—isometric view and (**d**) after lowering—isometric view.

Figure 12 shows loading of the 3D model of the Moste Dam in the case of full water reservoir and after lowering the water level of the reservoir, while Figure 13 shows the calculated horizontal displacements of the 3D model of the Moste Dam in both mentioned cases (the displacement scale is significantly larger than the scale in which the dam is shown; the magnification factor of 5000 was considered for displacements). The results of calculations of horizontal displacements on the 3D model show similar results as those obtained with the 2D model (Section 3.1.3). In the case of full water reservoir, the upper part of the dam moves downstream, while after lowering the water level of the reservoir, the upper part of the dam moves in the opposite direction (the calculated upstream horizontal displacement, due to the lowering of the water level, was 0.50 mm at the top of the vertical shaft; Table 6).

#### 3.2.3. Comparison of Calculated Horizontal Displacements

Table 7 shows the results of displacements of the 3D model of the dam according to two types of prescribed boundary conditions (variant A and variant B) and two differently dense meshes (29,960 and 72,000 finite elements). A comparison of the results of calculations shows that the mesh density has a small effect on the value of the calculated displacements in the stream direction, while the influence of the prescribed boundary conditions (or prescribed displacements) is very large. The results confirm that the best displacement value is obtained in the previously described case (Section 3.2.2), when the model of the dam is fixedly supported at the bottom, while on the sides of the dam only displacement in the stream direction is allowed.

It is understandable that such small displacements are negligible for this practical case. Exact values are given to compare the results of measurements with the results of calculations. The reason for the small displacements is a great rigidity of the arch-gravity Moste Dam, which, however, is the only dam in Slovenia where the experimental work can be carried out. In the case of less rigid and much higher dams (i.e., very high arch dam structures; e.g., up to 300 m), the measured displacements due to the changes in hydrostatic pressure would be significantly greater.

It has been shown that the consideration of changes in hydrostatic pressure on the dam is very important, as it represents one of the three parameters (in addition to seasonal temperature changes and time effects) that affect the horizontal displacements of a concrete dam over a long time period. In the case of a rapid lowering of the water level of the reservoir (when the influence of the other two factors on displacements is negligible), the horizontal displacements due to the reduction of hydrostatic pressure, which occur in a very short time period, can be exactly determined.

It has also been shown that in computational 3D analyses it is necessary to prepare a very accurate geometric model of the dam structure, using precise measurements (e.g., TLS measurements) at very low water level of the reservoir, which allows exact calculation of deformation behavior of the constructed engineering structure.

## 4. Conclusions

In this paper, the horizontal displacements of the large arch-gravity concrete dam were analyzed. A sophisticated automated system for the measurements of horizontal displacements of the upper part of the discussed dam was first established in 1998 and then re-established in 2013, when the pre-existing non-operating system was repaired (i.e., the three faulty inductive motion sensors were replaced with the new ones). Two-dimensional (2D) and three-dimensional (3D) analyses of dam behavior, taking into account the active earth pressures and the hydrostatic load on the upstream side of the dam and the passive earth pressures and the hydrostatic load on the downstream side of the dam, using the FEM-based computer program DIANA, were performed. The influence of lowering the water level of the reservoir by 6.2 m, on the horizontal displacements of the upper part of the dam, at stationary temperature conditions, was investigated. It was found that the results of the performed 2D and 3D FEM analyses matched very well the result of experimentally determined measurement of horizontal displacements (which was 0.48 mm in the upstream direction) that was obtained using a hanging pendulum. An additional comparison of the results of 3D calculations showed that the finite element mesh density had a small effect on the calculated horizontal displacements, while the influence of the prescribed boundary conditions is very large. The results confirm that in the 3D model of the dam, the best value of horizontal displacement is obtained when the model is fixedly supported at the bottom, while on the sides of the dam only displacement in the stream direction is allowed.

## Figures and Tables

**Table 1:** Main characteristics of the Moste Dam.

Characteristic	Unit	Value
Structural height	m	59.80
Crest length	m	72.00
Dam volume	m^3^	42,000
Crest altitude (steel gates)	m a.s.l.	524.75
Crest altitude (concrete)	m a.s.l.	523.50
Operational water level	m a.s.l.	518.00–524.75
Reservoir capacity	m^3^	6,240,000
Reservoir surface area	m^2^	620,000
Reservoir length	km	5.00
Catchment area	km^2^	325
Geographical latitude (North)	°	46.41
Azimuth (downstream side)	°	186

**Table 2:** Mechanical properties of the mass concrete.

Material Property	Unit	Value
Density (*ρ*)	kg/m^3^	2400
Elastic modulus (*E*)	GPa	30
Poisson’s ratio (*ν*)	/	0.2
Thermal expansion coefficient (*α_T_*)	K^−1^	10^−5^
Compressive strength (*f_c_*)	MPa	30 ^1^
Tensile strength (*f_t_*)	MPa	3 ^2^

^1^ Estimated compressive strength of concrete. ^2^ 10% of the estimated compressive strength of concrete.

**Table 3:** Material properties of the sediments and of the rock.

Material Property	Unit	Sediments ^1^	Rock ^2^
Density (*ρ*)	kg/m^3^	1800	2600
Effective friction angle (*φ’*)	°	23	58 ^3^
Effective cohesion (*c’*)	kPa	0	0

^1^ Lenart and Likar [45]. ^2^ Koprivec et al. [42]; Geršak [46]. ^3^ Equivalent friction angle determined from Hoek-Brown classification [47].

**Table 4:** Total active earth pressures acting on the upstream side of the dam, for two different water levels of the reservoir.

Height (m)	Medium	Active Pressure 1 ^1^ (Pa)	Active Pressure 2 ^2^ (Pa)	Difference (Pa)
50.20		10,693	0	10,693
45.09	Water	60,822	0	60,822
22.00		287,335	226,513	60,822
22.00	Sediments	287,335	226,513	60,822
12.00		419,816	358,994	60,822
12.00	Rock	391,888	331,066	60,822
0.00		525,095	464,273	60,822

^1^ Before lowering of the water level. ^2^ After lowering of the water level.

**Table 5:** Total passive earth pressures acting on the downstream side of the dam.

Height (m)	Medium	Passive Pressure ^1^ (Pa)
8.70	Water	0
6.00		26,487
6.00	Rock	26,487
0.00		1,230,721

^1^ The same all the time.

**Table 6:** Comparison of the measured and calculated displacement of the dam in the upstream direction, corresponding to the top of the shaft, due to the change in hydrostatic pressure.

Upstream Displacement (mm)		
Measured	Calculated (2D)	Calculated (3D)
0.48 ^1^	0.49 ^1^	0.50

^1^ See Section 3.1.2 and Section 3.1.3.

**Table 7:** Comparison of the calculated (3D) displacement of the dam in the upstream direction, corresponding to the top of the shaft, due to the change in hydrostatic pressure.

Boundary Conditions	Mesh Density (Number of Finite Elements)	Upstream Displacement (mm)
Variant A ^1^	29,960	0.08
	72,000	0.08
Variant B ^2^	29,960	0.50
	72,000	0.50

^1^ Below: fixed nodes; both sides: fixed nodes. ^2^ Below: fixed nodes; both sides: nodes movable only in the stream direction.

## Data Availability

The data presented in this study are available on request from the author. The data are not publicly available due to the requirements of the company Savske elektrarne Ljubljana Ltd, Medvode, Slovenia.
